# MRI in Oral Tongue Squamous Cell Carcinoma: A Radiomic Approach in the Local Recurrence Evaluation

**DOI:** 10.3390/curroncol32020116

**Published:** 2025-02-18

**Authors:** Antonello Vidiri, Vincenzo Dolcetti, Francesco Mazzola, Sonia Lucchese, Francesca Laganaro, Francesca Piludu, Raul Pellini, Renato Covello, Simona Marzi

**Affiliations:** 1 Radiology Unit, IRCCS Regina Elena National Cancer Institute, Via Elio Chianesi 53, 00144 Rome, Italy; antonello.vidiri@ifo.it (A.V.); francesca.laganaro@ifo.it (F.L.); francesca.piludu@ifo.it (F.P.); 2 Department of Radiological, Oncological and Pathological Sciences, Sapienza University of Rome, Policlinico Umberto I, Viale del Policlinico 155, 00161 Rome, Italy; vincenzo.dolcetti@uniroma1.it; 3 Otolaryngology and Head and Neck Surgery, IRCCS Regina Elena National Cancer Institute, Via Elio Chianesi 53, 00144 Rome, Italy; francesco.mazzola@ifo.it (F.M.); raul.pellini@ifo.it (R.P.); 4 Pathology Unit, IRCCS Regina Elena National Cancer Institute, Via Elio Chianesi 53, 00144 Rome, Italy; renato.covello@ifo.it; 5 Medical Physics Laboratory, IRCCS Regina Elena National Cancer Institute, Via Elio Chianesi 53, 00144 Rome, Italy; simona.marzi@ifo.it

**Keywords:** oral tongue squamous cell carcinoma (OTSCC), radiomics, machine learning (ML), MRI-based models, loco-regional recurrence, imaging biomarkers, precision oncology

## Abstract

(1) Background: Oral tongue squamous cell carcinoma (OTSCC) is a prevalent malignancy with high loco-regional recurrence. Advanced imaging biomarkers are critical for stratifying patients at a high risk of recurrence. This study aimed to develop MRI-based radiomic models to predict loco-regional recurrence in OTSCC patients undergoing surgery. (2) Methods: We retrospectively selected 92 patients with OTSCC who underwent MRI, followed by surgery and cervical lymphadenectomy. A total of 31 patients suffered from a loco-regional recurrence. Radiomic features were extracted from preoperative post-contrast high-resolution MRI and integrated with clinical and pathological data to develop predictive models, including radiomic-only and combined radiomic–clinical approaches, trained and validated with stratified data splitting. (3) Results: Textural features, such as those derived from the Gray-Level Size-Zone Matrix, Gray-Level Dependence Matrix, and Gray-Level Run-Length Matrix, showed significant associations with recurrence. The radiomic-only model achieved an accuracy of 0.79 (95% confidence interval: 0.69, 0.87) and 0.74 (95% CI: 0.54, 0.89) in the training and validation set, respectively. Combined radiomic and clinical models, incorporating features like the pathological depth of invasion and lymph node status, provided comparable diagnostic performances. (4) Conclusions: MRI-based radiomic models demonstrated the potential for predicting loco-regional recurrence, highlighting their increasingly important role in advancing precision oncology for OTSCC.

## 1. Introduction

Oral tongue squamous cell carcinoma (OTSCC) is the most common malignancy of the oral cavity (OC), accounting for about half of all instances of head and neck squamous cell carcinoma (SCC) [1]. Surgery is the primary treatment, followed by adjuvant radiotherapy +/− chemotherapy in the presence of negative pathological prognostic factors, such as a residual tumor, lymphovascular infiltration, or lymph node metastases with extracapsular extension (ENE) [2,3,4]. Immunotherapy is reserved for cases of recurrence (local or distant), refractoriness to platinum-based treatment, and tumor expression of a PD-L1 combined positive score > 1 [5]. Despite the use of combined therapies, the 5-year survival rate remains unsatisfactory (50–60%), and the frequency of loco-regional recurrence is high (approximately 40%) [6,7,8,9,10,11]. Among the most significant negative prognostic factors, the presence of lymph node metastases with ENE, positive resection margins, a greater depth of invasion (DOI) [8,10,12,13,14,15], and quantitative lymph node burden [16] have been demonstrated to be relevant negative prognostic factors. Currently, magnetic resonance imaging (MRI) is used for the loco-regional staging of tongue tumors [2], thanks to the high contrast resolution, which permits the obtainment of an optimal evaluation of the submucosal spread; for this reason, MRI represents a guide to surgical planning. Computed tomography (CT) and positron emission tomography (PET)–CT are techniques that can be used in the staging of oral tongue cancer. PET/CT represents a valuable alternative or complementary technique to MRI because of its high negative predictive value in the lymph node evaluation and proven benefits in post-therapy assessment [17,18]. At the same time, CT is useful in evaluating mandibular involvement (cortical infiltration) and in patients with contraindications to MRI [19].

Additional biomarkers are needed for imaging evaluation to stratify the patients and obtain personalized therapy. Regional recurrence is the most common cause of failure for OTSCC [20,21]; for this reason, identifying patients at risk of recurrence before primary surgery is very important to guide the treatment plan. Histopathological factors, through the biopsy before the surgery, are used for OTSCC diagnosis and prognosis. Still, the limitation of biopsy is that it may not capture the heterogeneity of the tumor due to a sampling bias. An emerging field of precision medicine is radiomics [22], which enables the extraction of quantitative features, such as texture, shape, and intensity, from radiological images. This large amount of quantitative data can be incorporated into machine learning (ML) models to better describe the tumor phenotype [23]. Radiomics provides a non-invasive, cost-effective, and reproducible approach to analyzing tumor heterogeneity, and can potentially improve the quality of tumor treatment within the framework of precision medicine and personalized care. Additionally, it has demonstrated promising prognostic value by quantifying the imaging features related to the entire tumor heterogeneity, which has been linked to adverse outcomes such as recurrence and poor survival [24,25].

This study aims to develop an MRI-based radiomic model to predict loco-regional recurrence from a retrospective setting in patients affected by OTSCC who underwent surgery.

## 2. Materials and Methods

### 2.1. Patient Population

We performed a retrospective radiomic analysis on the preoperative MRI of patients with OTSCC surgically treated at the Regina Elena National Cancer Institute.

This single-institution retrospective study was approved by the institutional ethics committee (RS1834/23). The requirement for obtaining written informed consent was waived due to the retrospective nature of this study.

Inclusion criteria were the diagnosis of OTSCC, surgery performed between 2014 and 2023, preoperative MRI (within 2 weeks before surgery), the presence of a tumor that could be measured on MRI, a pathological depth of invasion (pDOI) measurement in the histopathological report, and clinical information about the follow-up. The exclusion criteria were preoperative chemo-radiotherapy, classification as T4a for the mandibular infiltration, poor MRI quality because of motion artifacts and/or image distortion due to dental implants, and inadequate follow-up information (irretrievable medical information data).

All of the patients were staged and restaged according to the criteria outlined in the 8th edition of the AJCC TNM classification, which includes DOI as a crucial parameter for assessing the tumor extent and prognosis. For all cases, both the radiological DOI (rDOI) and pathological DOI (pDOI) were measured. The rDOI was determined using T1-weighted MRI sequences after the administration of the contrast medium. Specifically, the invasive portion of the tumor was measured by drawing a perpendicular line from a reference line, which was defined by the tumor surface and the normal mucosal surface on both sides, to the deepest point of the tumor, as described in the literature [19].

Data on alcohol consumption and smoking status were collected for all patients. Patients were classified as exposed or non-exposed based on alcohol consumption, and as non-smokers, current smokers, or former smokers based on smoking status. The type of surgical procedure was also recorded, distinguishing between transoral resection and the pull-through approach, both performed with concurrent neck dissection. These data are summarized in Table 1.

During follow-up, patients underwent periodic clinical and imaging evaluations with MRI and PET-CT; cases of loco-regional recurrence identified during these assessments were confirmed through biopsy.

### 2.2. MR Imaging Protocol

All of the patients underwent MRI examinations on 1.5 T (Optima MR 450w, GE Healthcare, Milwaukee, WI, USA) or 3 T (Discovery MR 750w, GE Healthcare, Milwaukee, WI, USA) scan systems using a 24- and 16-channel receive-only RF head–neck coil. The protocol included T2-weighted fast spin-echo coronal images (slice thickness, 4 mm), axial T2-weighted fast spin-echo images (slice thickness, 3 mm), and pre-contrast axial T1-weighted images (slice thickness, 3 mm) from the skull base to the thoracic inlet. Diffusion-weighted imaging (DWI) was also acquired using the following three b values: 0 s/mm^2^, 500 s/mm, and 1000 s/mm^2^ (slice thickness, 3 mm). A contrast-enhanced dynamic multiphase sequence was obtained using an axial T1-weighted fast-spoiled gradient echo sequence (FSPGR) after injecting a gadopentetate dimeglumine contrast agent at 0.1 mmol/kg body weight, including one pre-contrast volume and four post-contrast volumes, acquired with a temporal resolution of 31 s and a total scan duration of 2 min and 40 s, and the following parameters: acquisition matrix, 320 × 256; field of view, 25.6 × 25.6 cm; TR/TE, 5.9–6.6/2.1–2.9 ms; flip angle, 12°; slice thickness, 2 mm; slice spacing 1 mm. Alternatively, a dynamic contrast-enhanced (DCE) sequence was acquired using a 3D fast-spoiled gradient echo sequence with the following parameters: acquisition matrix, 162 × 150; field of view, 25 × 25 cm; TR/TE, 4.7/1.07 ms; flip angle, 30°; slice thickness, 4.4 mm; bandwidth, 390 Hz/pixel. During the passage of the contrast agent, 70 multiple volumes were acquired with a temporal resolution of 5 s and a total scan duration of 5 min 50 s. After three volumes, the contrast agent (0.1 mmol/kg body weight of gadopentetate dimeglumine) was intravenously injected at a rate of 3 mL/s.

Finally, FSPGR T1-weighted images in the axial and coronal planes were also acquired.

### 2.3. Volume Segmentation

To capture the peak of the contrast enhancement and better visualize the lesion, a *difference* volume was created based on a pre-contrast and a post-contrast volume. This *difference* volume was used to delineate the entire lesion, slice by slice, by an expert Head and Neck (HN) radiologist via semi-automatic tools in 3D Slicer software (version 5.6.2) [26]. Precisely, for patients who underwent a dynamic multiphase sequence, the post-contrast volume corresponded to the 3rd dynamic phase, while, for patients undergoing a DCE-MRI, the 17th dynamic volume was used to obtain comparable post-contrast interval times. This *difference* volume was created after co-registering the two volumes through (1) an affine transformation, followed by (2) a B-Spline deformable transformation. This two-step process allowed us to correct the possible global motion between the two image sets and to address local distortions caused by breathing, swallowing, and jaw movements, respectively [27].

### 2.4. Features Extraction

Before feature extraction, the *difference* volume was resampled to an isotropic voxel of a size of 1 mm and discretized with a bin width of 25. One hundred and seven features were extracted from the lesion using the 3D Slicer Radiomics Extension, built upon the PyRadiomics Python package (version 3.1.0). A comprehensive list of these features, including Shape (n = 14), First Order (n = 18), and Texture features (n = 75), is described in Supplementary Table S1. The ComBat method was applied using the neuroCombat package (version 1.0.13) in RStudio software (version 2024.04.2) [28] to harmonize the features across both different acquisition modalities and scanners. Specifically, the following three distinct patient groups were considered for harmonization: (1) patients undergoing a contrast-enhanced dynamic sequence at 1.5 T, (2) patients undergoing a contrast-enhanced dynamic sequence at 3 T, and (3) patients undergoing DCE-MRI at 3 T.

### 2.5. ML Modeling

Before model building, the radiomic features underwent some standardized pre-processing steps. These included the removal of outliers to exclude extreme values that could skew the analysis, the imputation of missing data, and Z-score normalization, which standardized features to a mean of zero and a standard deviation of one to obtain the same numerical range across the variables. Features strongly correlated with the lesion volume (i.e., Spearman’s correlation coefficient Rho ≥ 0.8) were adjusted by normalizing their values to the lesion volume so to eliminate size-dependent effects.

The following five predictive models for loco-regional recurrence were developed: (1) a pre-treatment clinical model, (2) a post-treatment clinical model, (3) a radiomic model, (4) a combined model integrating radiomic features with pre-surgical clinical data, and (5) a combined model integrating radiomic features with post-surgical clinical data.

To reduce the dimensionality of the potential predictors, the Adaptive Boosting (AdaBoost) classification algorithm was applied to rank the variables by importance score for each proposed model. Due to the limited sample size and to mitigate model overfitting, the top 10 features were selected for each model, and the best combination of 3 predictors among these 10 features was identified to build the final models.

Before model development, the dataset was randomly split into a training set (70%, n = 65) and a validation set (30%, n = 27), using a stratified splitting procedure to preserve the class distribution (recurrence/no recurrence) across sets.

To address the class imbalance, the Synthetic Minority Over-sampling Technique (SMOTE) was applied to the training set, generating synthetic instances for the minority class. SMOTE was implemented using the RSBID package (version 0.0.2.0000) in RStudio software [29], resulting in an augmented training set of 86 samples. Several machine learning algorithms were employed and compared, including Decision Trees, Discriminant Analysis, Logistic Regression, Naive Bayes, the Support Vector Machine (SVM), K-Nearest Neighbor, and Ensemble Classifiers. Hyperparameter optimization was conducted through stratified five-fold cross-validation to further minimize overfitting and identify the best-performing models. Model performance was assessed in terms of the accuracy, sensitivity, specificity, and area under the receiver operating characteristic curve (AUC), with the AUC calculated with a 95% bootstrap confidence interval from 1000 samples. Pairwise model comparisons were conducted using the mid-*p*-value McNemar test to evaluate the prediction accuracies. All of the analyses and model development were performed using the Statistics and Machine Learning MATLAB Toolbox (MATLAB Release R2021b, MathWorks Inch; Natick, MA, USA).

### 2.6. Statistical Analysis

Statistical tests were conducted to explore the relationship between the radiomic features and recurrence status. The Mann–Whitney U test was employed for each feature to assess the statistical significance with a *p*-value threshold of <0.15. Features with strong intercorrelation (Spearman’s correlation coefficient Rho > 0.80) were filtered to reduce redundancy, retaining only the feature most significantly associated with recurrence. Clinico-pathological features, both pre- and post-treatment, were similarly analyzed for associations with recurrence. Continuous variables were analyzed using the Mann–Whitney U test, while categorical variables were assessed with the Chi-square test. Associations between variables were examined according to the following data types: Spearman correlation for continuous variables, Chi-square for categorical variables, and the Kruskal–Wallis test as appropriate. Data distributions were visualized using boxplots. Univariate Cox regression analysis was used to evaluate the potential of the selected variables for the classification models as predictors for loco-regional recurrence-free survival (LRRFS). Multivariate Cox regression analysis was used to identify independent prognostic factors for LRRFS using the stepwise method. The hazard ratio (HR) and its relative 95% confidence intervals (95% CIs) were calculated.

LRRFS was analyzed using the Kaplan–Meier product-limit method, and the log-rank test was used to test for potential differences between the curves.

All of the statistical analyses were performed in MATLAB (Release R2021b, MathWorks Inch; Natick, MA, USA).

## 3. Results

A total of 92 surgically treated OTSCC patients met the inclusion criteria for this study, including 31 with loco-regional recurrence and 61 without loco-regional recurrence. The anonymized database, including all relevant variables, has been provided in the Supplementary Materials (File S1).

The median follow-up time was 2.5 years (95% CI: 2.1–3.42). 

A total of 54 patients underwent contrast-enhanced dynamic sequences at 1.5T, 14 at 3T, and 24 at DCE-MRI at 3T. The effect of ComBat-based feature harmonization, applied to reduce the variability related to the different scanners and acquisition protocols, is shown in Supplementary Figure S1.

Patient and tumor characteristics of the entire patient population, and the training and validation cohorts, are reported in Table 1.

**Figure 1:**
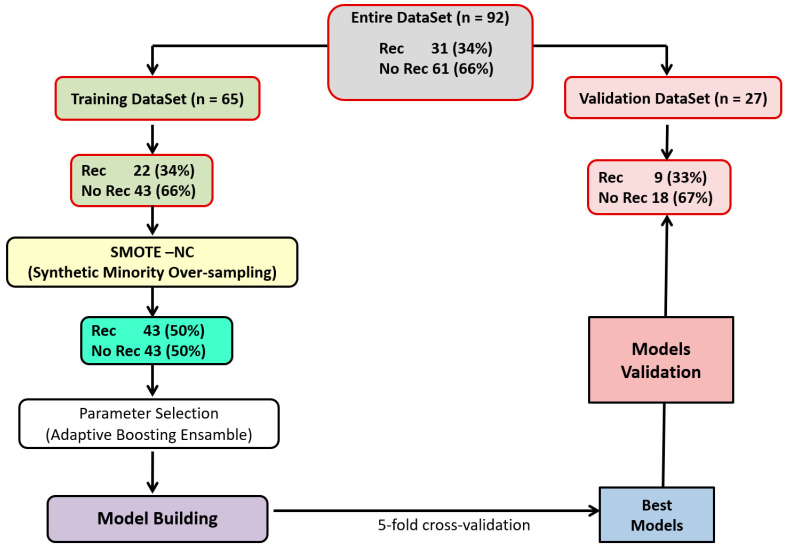
The flowchart illustrating the entire pipeline of the analyses to obtain the final models.

No significant differences were observed between the training and validation sets regarding patient and tumor characteristics. The entire pipeline of the analyses is illustrated in Figure 1.

### 3.1. ML Modeling

The variables retained after the feature selection process are shown in Supplementary Figure S2.

In the pre-treatment clinical model, the most relevant predictors identified were the MR-based radiological dimension, rDOI, and sex. In contrast, the pathological dimensions, pN, pDOI, and sex were selected as the top predictors for the post-treatment clinical model. Large-area high-gray-level emphasis (from GLSZM), large-dependence high-gray-level emphasis (from GLDM), and long-run emphasis (from GLRLM) were the most relevant predictors for the radiomic model. The best predictors in the combined model based on pre-surgical clinical data and radiomic features were the MR-based radiological dimensions, large-area high-gray-level emphasis, and long-run emphasis. In the combined model based on post-surgical clinical and radiomic data, the selected predictors were sex, large-area high-gray-level emphasis, and long-run emphasis. The diagnostic performances of the four proposed models are shown in Table 2, and the corresponding confusion matrices are illustrated in Supplementary Figure S3. Summary statistics of all predictors included in the final models are reported in Table 3 for patients with and without recurrence.

Even though it was not included in the final models, the pDOI showed a statistically significant difference (*p* = 0.028) between the two patient groups: the median was 13 mm (95% CI: 12–25) and 9 mm (95% CI: 7–12) for patients with and without recurrence, respectively. In contrast, the rDOI exhibited only a trend toward significance (*p* = 0.087), with a median of 13 mm (95% CI: 10–17) in patients with recurrence and 11 mm (95% CI: 8–13) in those without. A similar trend toward statistical significance was observed for alcohol consumption (*p* = 0.072) and the type of surgery performed (*p* = 0.102), though neither was included in the final models.

**Figure 2:**
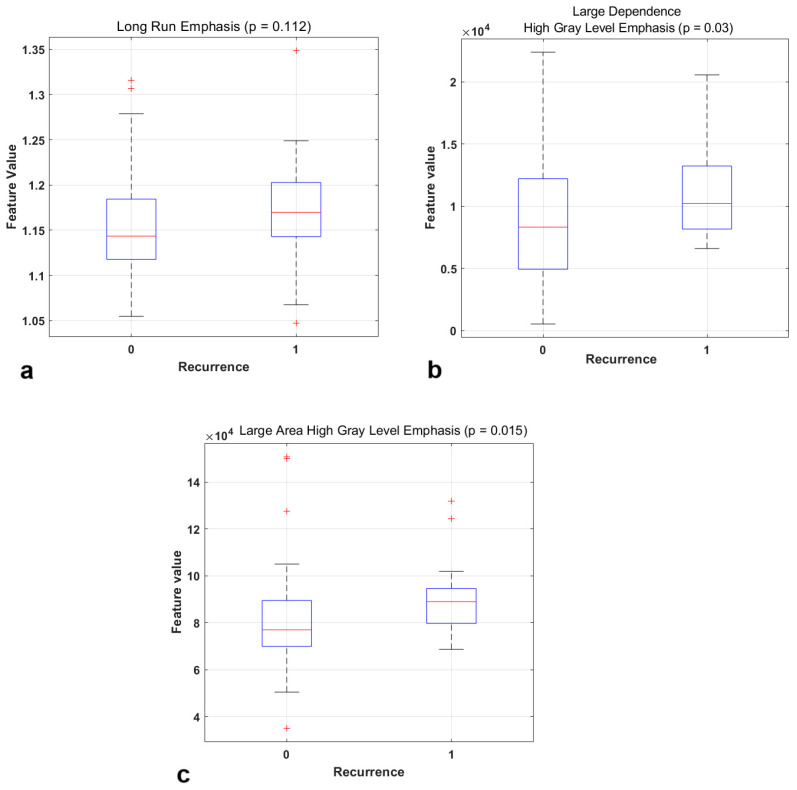
Boxplots of the most relevant radiomic features for predicting the recurrence and the corresponding *p*-value obtained from the Mann–Whitney U test: Long Run Emphasis (**a**), Large Dependence High Gray Level Emphasis (**b**), Large Area High Gray Level Emphasis (**c**).

Among the different machine ML algorithms, the SVM and the Ensemble of Decision Trees provided the best performances in terms of the accuracy and AUC. Specifically, the radiomic-only model, trained with an ensemble of Decision Trees, had the highest predictive performance for loco-regional recurrence, achieving an accuracy of 0.79 (95% CI: 0.69–0.87) and 0.74 (95% CI: 0.54–0.89) on the training set and on the validation set, respectively. The boxplots of the most relevant radiomic features included in this classification model are illustrated in Figure 2.

The pre-treatment clinical model exhibited poor accuracy, which was <0.60. In contrast, the best combined pre-treatment model, obtained from an ensemble of Decision Trees, achieved a good accuracy, reaching 0.77 (95% CI: 0.66–0.85) on the training set and 0.70 (95% CI: 0.50–0.86) on the validation set.

The best-performing classifier for the post-treatment clinical-only model was an SVM, with accuracies of 0.72 (95% CI: 0.61–0.81) and 0.67 (95% CI: 0.46–0.83) on the training and validation sets, respectively.

The combined post-treatment model, trained with an ensemble of Decision Trees, reached a good accuracy of 0.77 (95% CI: 0.66–0.85) and 0.74 (95% CI: 0.54–0.89) on the training set and the validation set, respectively. As assessed by McNemar’s test, no statistically significant differences in the performance among all of the proposed models were observed on either dataset (see Supplementary Table S2).

**Figure 3:**
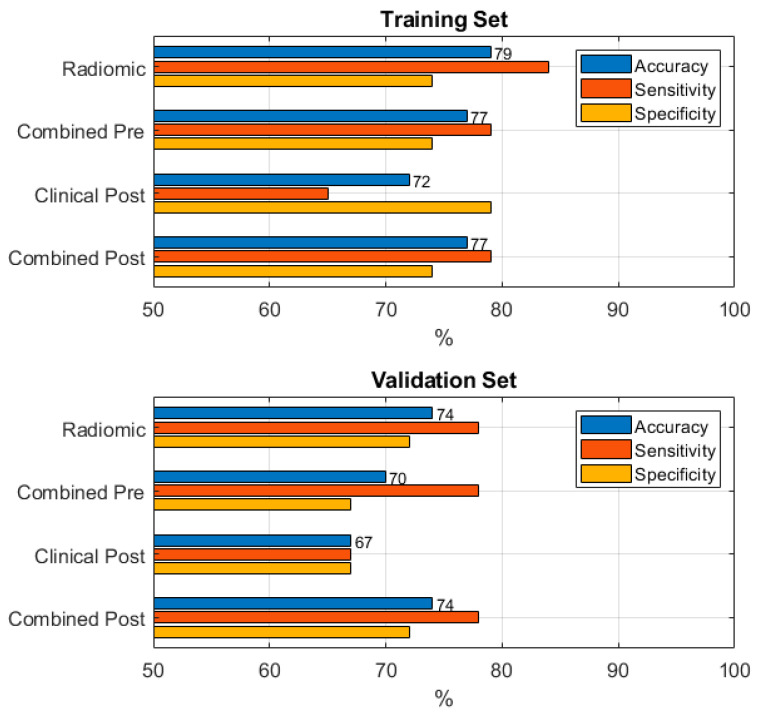
Comparison between the performances of the different models for predicting the loco-regional recurrence in training and validation sets.

A graphical overview of model performance is reported in Figure 3.

### 3.2. Illustrative Cases

**Figure 4:**
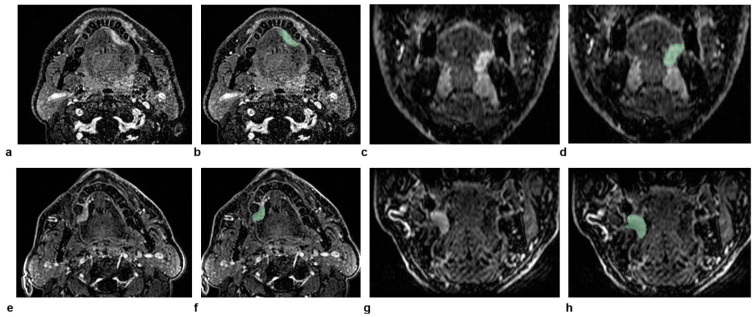
The figure shows dynamic high-spatial-resolution T1-weighted images after contrast medium administration in the axial and coronal planes of two patients with the same radiological staging (cT2N0) but different clinical outcomes, for whom the radiomic-only model and all combined models provided the correct predictions. In the top row (**a**–**d**), the images represent a 58-year-old male patient who did not experience a recurrence, with the corresponding delineated lesion outlined in green on the same planes (**b**,**d**). In the bottom row (**e**–**h**), the images represent a 71-year-old male patient with a recurrence. Similarly, the axial and coronal images (**e**,**g**) show the lesion after contrast medium administration, with the delineated lesion outlined in green (**f**,**h**).

Two representative patients with the same radiological staging (cT2N0) but different clinical outcomes are illustrated in Figure 4. The radiomic-only model and all of the combined models provided the correct predictions for both patients. This can be explained considering that the values of large-area high-gray-level emphasis, large-dependence high-gray-level emphasis, and long-run emphasis were all lower in the patient without recurrence compared to the patient with recurrence (6.6 × 10^4^, 4.7 × 10^3^, and 1.1 versus 1.0 × 10^5^, 1.5 × 10^4^, and 1.2, respectively).

### 3.3. Loco-Regional Recurrence-Free Survival Analysis

Exploratory analyses were performed to test the ability of the variables already selected for the classification models to also predict LRRFS.

The hazard ratios of the included variables are reported in Table 4: patients with a positive pN status had an increased risk of at least 3-fold of developing a loco-regional recurrence compared to patients with pN0; the radiological dimension of the lesion and rDOI were marginally statistically significant, showing a very small effect size; the type of surgery indicated that patients who underwent a transoral surgery plus neck dissection had a better outcome compared to patients receiving a pull-through surgery plus neck dissection, even though on the threshold of statistical significance. In the multivariate Cox regression analysis, only the pN status was retained as an independent predictor for LRRFS, while the radiological dimension of the lesion, rDOI, and type of surgery were removed.

**Figure 5:**
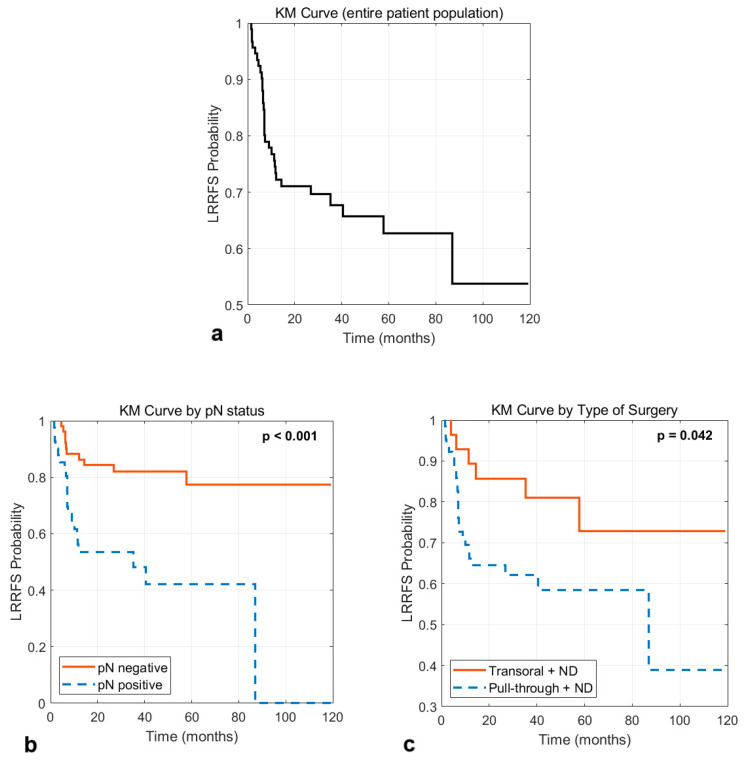
Kaplan–Meier survival curves for loco-regional recurrence-free survival (LRRFS) for the entire dataset (**a**), and by categorizing the patients based on pN status (**b**) and type of surgery (**c**). The *p*-values refer to the log-rank test for the pairwise comparison between groups.

The KM curve of the entire patient population and the KM curves obtained by categorizing the patients based on pN status and type of surgery are illustrated in Figure 5. The 5-year LRRFS probability for all patients was 62.7%; the 5-year LRRFS probability for patients with a negative and positive pN status was 77.4% and 42.1%, respectively (*p* < 0.001); the 5-year LRRFS probability for patients receiving transoral surgery plus neck dissection was 72.9% compared to 58.4% for patients receiving a pull-through surgery plus neck dissection (*p* = 0.042).

## 4. Discussion

Integrating advanced ML-driven methodologies into the clinical management of OTSCC has emerged as a promising avenue for enhancing the prognostic accuracy and personalizing treatment strategies in head and neck cancer, as supported by the recent literature [24,25,30,31].

Our findings emphasized the potential of radiomics, alone or combined with clinical and pathological features, to predict loco-regional recurrence, which remains a major clinical challenge in the management of OTSCC [24]. Radiomics is a powerful and non-invasive tool for analyzing medical images, which captures spatial heterogeneity and microstructural tissue characteristics from the entire lesion for a more comprehensive cancer characterization [22,23,24].

High-order textural features derived from high-resolution post-contrast MRI, together with pN, radiological or pathological tumor dimension, and sex, were found to be the most relevant predictors. Specifically, patients with loco-regional recurrence showed increased values of textural features—such as large-area high-gray-level emphasis (from GLSZM), large-dependence high-gray-level emphasis (from GLDM), and long-run emphasis (from GLRLM)—suggesting that their lesions were characterized by higher heterogeneity and tumor microenvironment complexity [32,33]. In fact, increased values of the large-area high-gray-level emphasis indicated larger areas with high-intensity gray levels that may represent well-organized tumor structures or more dense tumor sub-volumes; these regions could reflect biological conditions like hypoxia, aggressive growth, or resistance to treatment, potentially linked to an increased likelihood of loco-regional recurrence [34,35].

Similarly, large-dependence high-gray-level emphasis evaluated the dependency of high-gray-level intensities in neighboring pixels; a higher dependency might indicate a more structured tumor texture, potentially associated with more invasive margins. It might also suggest increased stromal involvement or denser tumor cell packing, both of which are linked to aggressive behavior and poor outcomes [34,36].

Lastly, an increased long-run emphasis is related to the continuity of pixel intensities over longer runs, highlighting macroscale homogeneity in the tumor tissue. This characteristic may correlate with uniformly growing tumor subregions, which are generally less responsive to treatments like chemotherapy or radiotherapy [32,33,36].

The use of clinical features alone did not result in a pre-treatment clinical model with an adequate profile in diagnostic accuracy (it was <0.6) and was not proposed as a possible model.

Even though statistically significant differences in the performance of all of the proposed models were not observed, the post-treatment clinical model provided lower performance than radiomic-only or combined models. This underscores the potential of radiomics to complement traditional clinical and pathological parameters in identifying high-risk patients, according to Fatapour et al. [24], who highlighted the importance of radiomic biomarkers in capturing tumor characteristics, which are often the precursors to recurrence.

This is also consistent with findings from Alkhadar et al. [37], who compared different ML models trained by various ML algorithms, such as SVM and Random Forests, to predict loco-regional recurrence in a large population with oral SCC. Similarly, Tseng et al. [38] and Tagliabue et al. [25] reported enhanced prognostic capabilities by integrating radiomic and clinical data, highlighting the synergistic value of multimodal approaches in oral cancer.

It is noteworthy that both the pDOI and rDOI showed a predictive ability to differentiate patients with and without recurrence in our patient population, even if they were not included in the final models by the ML-driven algorithms. This was probably due to their positive correlation with the pathological/radiological tumor dimension (which, instead, entered into the models); thus, they were excluded to reduce data redundancy and mitigate model overfitting. However, the important role of pDOI/rDOI as a predictor of loco-regional recurrence, cervical lymph node metastasis, and survival in tongue cancer was concordantly reported by several investigators, suggesting that it is a relevant parameter to be considered for prognostication models [13,27,39,40,41].

While the smoking status did not show a statistically significant difference between patients with and without recurrence, alcohol consumption and surgical approach showed a trend toward significance, even though they were not included in the final classification models. The tendency towards the statistical significance of the type of surgery, followed by its exclusion from the classification models, could be explained by its correlation with the T stage, which is determined by the pDOI and the pathological dimension. At the same time, patients receiving a pull-through surgery plus neck dissection showed a reduced 5-year LRRFS probability compared to patients receiving a transoral surgery plus neck dissection, which can be attributed to the largest tumor extents and more advanced-stage OTSCCs.

Concerning alcohol and smoking habits, their relationship with loco-regional recurrence is complex and may be influenced by multiple factors. While smoking and alcohol consumption are well-known risk factors in the pathogenesis of OCSCC, recent studies have indicated that non-smoking and non-drinking patients did not show a clear survival advantage [42]. Further research is needed to better understand the underlying biological mechanisms of loco-regional recurrence related to smoking and alcohol consumption in OCSCC, also taking into consideration independent biological factors, such as genetic and molecular patterns, and tumor microenvironment characteristics.

Additionally, sex was found to show a trend toward significance, indicating higher recurrence rates in women. This may be clinically relevant, and is consistent with the findings from Mossinelli et al. [30]. The authors reported poorer overall survival rates in female patients, suggesting potential biological or hormonal influences that warrant further investigation. These insights could guide more personalized follow-up protocols for female patients.

In the present study, we focused on classification models for predicting loco-regional recurrence, and we plan to investigate LRRFS and overall survival models after a longer follow-up. However, we preliminarily explored the potential of the selected variables for the classification models also to predict LRRFS. It emerged that the pN status, as reported in NCCN guidelines [43], was the only independent predictor for LRRFS, while no radiomic features were significant. This is also in agreement with the findings of Tam et al. [13] and Ganly et al. [15], which highlighted the crucial prognostic role of the pN status in the recurrence-free survival of patients with OTSCC, demonstrating that the presence of lymph node metastases was associated with a higher risk of recurrence and worse oncological outcomes. While the results of this study are promising, several challenges must be addressed for the clinical adoption of AI-driven tools in OTSCC management. Variability in imaging protocols, equipment, image post-processing, feature extraction/selection procedures, and approaches to different ML algorithms hinder the reproducibility. These issues underscore the need for standardized data collection and multicentric studies to improve the robustness and generalizability. Additionally, many studies, including this one, rely on retrospective, single-center data from small patient populations, which may not fully capture the diverse clinical presentations and increases the risk of selection bias. Moreover, integrating AI tools into clinical workflows requires compatibility with electronic health records, interoperability across healthcare systems, and secure data handling [40]. In this investigation, we used the ComBat method to harmonize the radiomic features and mitigate the impact of differences in acquisition modalities and scanners [44]. At the same time, we employed a five-fold cross-validation technique to reduce the risk of overfitting due to the small sample size, which may compromise the generalizability and reproducibility of the results [45].

Our study has several limitations. As mentioned, it is monocentric, retrospective, and based on a small sample size. Only a single expert HN radiologist performed the lesion segmentation via semi-automatic tools, and we did not evaluate the impact of inter- and intraoperator variability on the radiomic features. Furthermore, we did not explore the potential of all radiomic features, alone or in combination with clinical and pathological data, for the LRRFS and overall survival models, which will be addressed in future research after a long-term follow-up.

## 5. Conclusions

MRI-based radiomic models demonstrated the potential for predicting loco-regional recurrence in OTSCC, outperforming clinical-only models and providing fair-to-good accuracies in both training and validation sets.

High-order textural features derived from high-resolution post-contrast MRI, together with pN, radiological or pathological tumor dimension, and sex, were found to be the most relevant predictors. A larger dataset is needed to confirm our findings and evaluate their transportability on an external patient cohort.

## Figures and Tables

**Table 1:** Patients and tumor characteristics in the entire dataset, training set, and validation set.

	Entire Set (n = 92)	Training Set (n = 65)	Validation Set (n = 27)	*p*-Value
**Age (years)**	64.5 [60, 68.9]	66 [59.3, 69.9]	61 [54, 69]	0.425
**Sex**				0.820
Female	46 (50%)	32 (49.2%)	14 (51.9%)	
Male	46 (50%)	33 (50.8%)	13 (48.1%)	
**Smoking Status**				0.161
Non-smoker	40 (43.5%)	32 (49.2%)	8 (29.6%)	
Current smoker	32 (34.8%)	19 (29.2%)	13 (48.1%)	
Former smoker	20 (21.7%)	14 (21.5%)	6 (22.2%)	
**Alcohol Consumption**				0.699
No	69 (75%)	50 (76.9%)	19 (70.4%)	
Yes	22 (23.9%)	15 (23.1%)	7 (25.9%)	
Not reported	1 (1.1%)	0	1 (3.7%)	
**Type of Surgery**				0.545
Transoral + ND	28 (30.4%)	21 (32.3%)	7 (25.9%)	
Pull-through + ND	64 (69.6%)	44 (67.7%)	20 (74.1%)	
**Radiological Dimensions (mm)**	30 [25.1, 35]	30 [26, 35]	26 [19.9, 41]	0.857
**rDOI (mm)**	11 [9.2, 13]	11 [9, 13.9]	11 [9, 15.5]	0.777
**cT**				0.546
T1	8 (8.7%)	7 (10.8%)	1 (3.7%)	
T2	33 (35.9%)	23 (35.4%)	10 (37%)	
T3	51 (55.4%)	35 (53.8%)	16 (59.3%)	
**cN**				
N0	41 (44.6%)	28 (43.1%)	13 (48.1%)	
N1	21 (22.8%)	15 (23.1%)	6 (22.2%)	
N2b	15 (16.3%)	12 (18.5%)	3 (11.1%)	
N2c	3 (3.3%)	1 (1.5%)	2 (7.4%)	
N3b	12 (13%)	9 (13.8%)	3 (11.1%)	
**Pathological Dimensions (mm)**	25 [21.1, 30]	25 [20, 30]	25 [20, 32.1]	0.945
**pDOI (mm)**	11.5 [8, 12]	11 [8, 13]	12 [8, 15]	0.990
**pT**				0.366
T1	13 (14.1%)	11(16.9%)	2 (7.4%)	
T2	30 (32.6%)	19 (29.2%)	11 (40.7%)	
T3	49 (53.3%)	35 (53.8%)	14 (51.9%)	
**pN**				
N0	51 (55.4%)	35 (53.8%)	16 (59.3%)	
N1	9 (9.8%)	5 (7.7%)	4 (14.8%)	
N2a	2 (2.2%)	2 (3.1%)	0	
N2b	13 (14.1%)	11 (16.9%)	2 (7.4%)	
N2c	2 (2.2%)	1 (1.5%)	1 (3.7%)	
N3b	15 (16.3%)	11 (16.9%)	4 (14.8%)	
**Grow Pattern**				0.672
Expansive	28 (30.4%)	18 (27.7%)	10 (37%)	
Infiltrative	37 (40.2%)	27 (41.5%)	10 (37%)	
Mixed	27 (29.3%)	20 (30.8%)	7 (25.9%)	
**Grading**				
G1	4 (4.3%)	2 (3.1%)	2 (7.4%)	
G2	54 (58.7%)	38 (58.5%)	16 (59.3%)	
G3	34 (37%)	25 (38.5%)	9 (33.3%)	
**Lymphovascular Infiltration**				0.828 *
No	67 (72.8%)	49 (75.4%)	18 (66.7%)	
Yes	8 (8.7%)	5 (7.7%)	3 (11.1%)	
Not reported	17 (18.5%)	11 (16.9%)	6 (22.2%)	
**Perineural Infiltration**				0.353
No	35 (38%)	27 (41.5%)	8 (29.6%)	
Yes	40 (43.5%)	27 (41.5%)	13 (48.1%)	
Not reported	17 (18.5%)	11 (16.9%)	6 (22.2%)	
**Keratinization**				
Focal	18 (19.6%)	15 (23.1%)	3 (11.1%)	
No	7 (7.6%)	4 (6.2%)	3 (11.1%)	
Yes	63 (68.5%)	43 (66.2%)	20 (74.1%)	
Not reported	4 (4.3%)	3 (4.6%)	1 (3.7%)	
**Surgical Margins §**				
No	89 (96.7%)	-	-	
Yes	1 (1.1%)	-	-	
Not reported	2 (2.2%)	-	-	
**ENE**				0.888 *
No	78 (84.8%)	56 (86.2%)	22 (81.5%)	
Yes	13 (14.1%)	9 (13.8%)	4 (14.8%)	
Not reported	1 (1.1%)	0	1 (3.7%)	
**Micrometastases §**				
No	91 (98.9%)	-	-	
Yes	1 (1.09%)	-	-	
**Adjuvant treatment**				0.694
No	38 (41.3%)	26 (40%)	12 (44.4%)	
Yes	54 (58.7%)	39 (60%)	15 (55.6%)	
**Loco-regional Recurrences**				0.964
No	61 (66.3%)	43 (66.2%)	18 (66.7%)	
Yes	31 (33.7%)	22 (33.8%)	9 (33.3%)	
**Follow-up time (months)**	30.6 [25.5, 41.6]	29.5 [23, 43.6]	32.1 [22.8, 48.0]	0.874

Numerical variables are presented as median values with 95% confidence intervals; categorical variables are expressed as counts. No *p*-value is reported for categorical variables with insufficient class counts. * Yates’ correction to *p*-values was applied. § Variables excluded from the analyses due to class imbalance. Abbreviations: ND, neck dissection; rDOI, radiological depth of invasion; pDOI, pathological depth of invasion; ENE, extranodal extension.

**Table 2:** Performance of the prediction models for recurrence in the training and validation sets.

Model	AUC	Acc	Sens	Spec	PPV	NPV
**Radiomic** (1) Large-Area High-Gray-Level Emphasis (GLSZM) (2) Large-Dependence High-Gray-Level Emphasis (GLDM) (3) Long-Run Emphasis (GLRLM)	0.78 [0.66, 0.87]	0.79 [0.69, 0.87]	0.84 [0.69, 0.93]	0.74 [0.59, 0.86]	0.77 [0.66, 0.85]	0.82 [0.69, 0.90]
	**0.75** **[0.52, 0.89]**	**0.74** **[0.54, 0.89]**	**0.78** **[0.40, 0.97]**	**0.72** **[0.47, 0.90]**	**0.58** **[0.38, 0.76]**	**0.87** **[0.65, 0.96]**
**Combined** **Pre-treatment** (1) Radiological Dimensions (2) Large-Area High-Gray-Level Emphasis (GLSZM) (3) Long-Run Emphasis (GLRLM)	0.82 [0.71, 0.90]	0.77 [0.66, 0.85]	0.79 [0.64, 0.90]	0.74 [0.59, 0.86]	0.76 [0.64, 0.84]	0.78 [0.66, 0.87]
	**0.72** **[0.51, 0.86]**	**0.70** **[0.50, 0.86]**	**0.78** **[0.40, 0.87]**	**0.67** **[0.41, 0.87]**	**0.54** **[0.36, 0.71]**	**0.86** **[0.63. 0.96]**
**Post-treatment** **Clinical** (1) Pathological Dimensions (2) pN (3) Sex	0.74 [0.62, 0.83]	0.72 [0.61, 0.81]	0.65 [0.49, 0.79]	0.79 [0.64, 0.90]	0.76 [0.63, 0.85]	0.69 [0.59, 0.78]
	**0.67** **[0.45, 0.83]**	**0.67** **[0.46, 0.83]**	**0.67** **[0.30, 0.93]**	**0.67** **[0.41, 0.87]**	**0.50** **[0.31, 0.69]**	**0.80** **[0.60, 0.91]**
**Combined** **Post-treatment** (1) Sex (2) Large-Area High-Gray-Level Emphasis (GLSZM) (3) Long-Run Emphasis (GLRLM)	0.82 [0.71, 0.90]	0.77 [0.66, 0.85]	0.79 [0.64, 0.90]	0.74 [0.59, 0.86]	0.76 [0.64, 0.84]	0.78 [0.66, 0.87]
	**0.75** **[0.49, 0.89]**	**0.74** **[0.54, 0.89]**	**0.78** **[0.40, 0.97]**	**0.72** **[0.47, 0.90]**	**0.58** **[0.38, 0.76]**	**0.87** **[0.65, 0.96]**

Data in **bold** refer to the validation set. Performance values are reported with their corresponding 95% confidence intervals. Abbreviations: GLSZM, Gray-Level Size-Zone Matrix; GLDM, Gray-Level Dependence Matrix; GLRLM, Gray-Level Run-Length Matrix; AUC, area under the receiver operating characteristic curve; PPV, positive predictive value; NPV, negative predictive value.

**Table 3:** Summary statistics of the best predictors included in the final models.

	No Recurrence	Recurrence	
Numerical Predictor	Median [95% CI]	Median [95% CI]	*p*-Value
Large-Area High-Gray-Level Emphasis (GLSZM)	7.7 [7.2, 8.4] × 10^4^	8.7 [7.9, 9.4] × 10^4^	0.015
Large-Dependence High-Gray-Level Emphasis (GLDM)	0.8 [0.7, 1.0] × 10^4^	1.0 [0.9, 1.2] × 10^4^	0.030
Long-Run Emphasis (GLRLM)	1.14 [1.13, 1.16]	1.17 [1.15, 1.20]	0.112
Radiological Dimensions (mm)	26 [21, 35]	33 [27, 40]	0.111
Pathological Dimensions (mm)	24 [20, 25]	30 [25, 35]	0.067
Categorical Predictor	Counts	Counts	*p*-Value
**Sex**			0.123
Female	27	19	
Male	34	12	
**pN**			
N0	41	10	
N1	2	7	
N2a	2	0	
N2b	7	6	
N2c	0	2	
N3b	9	6	

Numerical variables are presented as median values with corresponding 95% confidence intervals, while categorical variables are expressed as counts. The *p*-value for pN is omitted due to insufficient class frequencies. Abbreviations: CI, confidence interval; GLSZM, Gray-Level Size-Zone Matrix; GLDM, Gray-Level Dependence Matrix; GLRLM, Gray-Level Run-Length Matrix.

**Table 4:** Hazard ratios from the univariate Cox analysis including predictors selected for the classification models.

Predictor	HR [95% CI]	*p*-Value
**pN**		
pN0	Referent	
pN1	4.61 [1.75, 12.16]	0.002
pN2	4.22 [1.63, 10.95]	0.003
pN3	3.03 [1.09, 8.42]	0.034
**Radiological Dimensions**	1.03 [1.00, 1.05]	0.041
**rDOI**	1.05 [1.00, 1.09]	0.041
**Type of Surgery**		
Pull-through + ND	Referent	
Transoral + ND	0.41 [0.17, 1.00]	0.049
**pDOI**	NS	NS
**Pathological Dimensions**	NS	NS
**Large-Dependence High-Gray-Level Emphasis**	NS	NS
**Large-Area High-Gray-Level Emphasis**	NS	NS
**Long-Run Emphasis**	NS	NS
**Alcohol Consumption**	NS	NS
**Sex**	NS	NS

Abbreviations: HR, hazard ratio; CI, confidence interval; rDOI, radiological depth of invasion; ND, neck dissection; pDOI, pathological depth of invasion; NS, not significant.

## Data Availability

The original contributions presented in this study are included in the article/Supplementary Materials. Further inquiries can be directed to the corresponding author.

## Supplementary Materials

The following supporting information can be downloaded at: https://www.mdpi.com/article/10.3390/curroncol32020116/s1, File S1: Database Anonymized; Figure S1: Effect of ComBat-based feature harmonization; Figure S2: Feature selection using the Adaptive Boosting (AdaBoost) classification algorithm; Figure S3: Confusion matrices of the models; Table S1: List of radiomic features included in the study; Table S2: Comparison of model performances in the training and validation sets.

## Abbreviations

The following abbreviations are used in this manuscript:

OTSCC	Oral Tongue Squamous Cell Carcinoma
MRI	Magnetic Resonance Imaging
GLSZM	Gray-Level Size-Zone Matrix
GLDM	Gray-Level Dependence Matrix
GLRLM	Gray-Level Run-Length Matrix
pDOI	Pathological Depth of Invasion
rDOI	Radiological Depth of Invasion
ML	Machine Learning
OC	Oral Cavity
SCC	Squamous Cell Carcinoma
ENE	Extranodal Extension
PD-L1	Programmed Death-Ligand 1
DOI	Depth of Invasion
CT	Computer Tomography
PET-CT	Positron Emission Tomography/Computed Tomography
AI	Artificial Intelligence
FSPGR	Fast-Spoiled Gradient Echo
DCE	Dynamic Contrast-Enhanced
TR	Repetition Time
TE	Echo Time
HN	Head and Neck
SMOTE	Synthetic Minority Over-sampling Technique
RSBID	Resampling Strategies for Binary Imbalanced Datasets
AUC	Area Under the Curve
CI	Confidence Interval
SVM	Support Vector Machine
OCSCC	Oral Cavity Squamous Cell Carcinoma
