# Supplementary material for: MRI in Oral Tongue Squamous Cell Carcinoma: A Radiomic Approach in the Local Recurrence Evaluation
Source: Curr Oncol. 2025 Feb 18;32(2):116. doi: 10.3390/curroncol32020116 (PMC11854587; doi:10.3390/curroncol32020116)
Supplement: Supplementary file 1 [file curroncol-32-00116-s001.zip › Supplementary Materials.pdf]

Content:

Supplementary Tables

- ✓ **Supplementary Table S1:** List of radiomic features included in the study
- ✓ **Supplementary Table S2:** Comparison of model performances in the training and validation sets

Supplementary Figures

- ✓ **Supplementary Figure S1:** Effect of ComBat-based feature harmonization
- ✓ **Supplementary Figure S2:** Feature selection using the Adaptive Boosting (AdaBoost) classification algorithm
- ✓ **Supplementary Figure S3**: Confusion matrices of the models

Supplementary Table S1. Complete List of the Extracted Radiomic Features.

Feature Family (count)	Feature Name
Shape (14)	Elongation
	Flatness
	Least Axis Length
	Major Axis Length
	Maximum 2D Diameter Column
	Maximum 2D Diameter Row
	Maximum 2D Diameter Slice
	Maximum 3D Diameter
	Mesh Volume
	Minor Axis Length
	Sphericity
	Surface Area
	Surface Volume Ratio
	Voxel Volume
First Order (18)	10th Percentile
	90th Percentile
	Energy
	Entropy
	Interquartile Range
	Kurtosis
	Maximum
	Mean Absolute Deviation
	Mean
	Median
	Minimum
	Range

	Robust Mean Absolute Deviation
	Root Mean Squared
	Skewness
	Total Energy
	Uniformity
	Variance
Gray-level co-occurrence matrix (24)	Autocorrelation
	Cluster Prominence
	Cluster Shade
	Cluster Tendency
	Contrast
	Correlation
	Difference Average
	Difference Entropy
	Difference Variance
	Id
	Idm
	Idmn
	Idn
	Imc1
	Imc2
	Inverse Variance
	Joint Average
	Joint Energy
	Joint Entropy
	MCC
	Maximum Probability
	Sum Average

	Sum Entropy
	Sum Squares
Gray-Level Dependence Matrix (14)	Dependence Entropy
	Dependence Non Uniformity
	Dependence Non Uniformity Normalized
	Dependence Variance
	Gray Level Non Uniformity
	Gray Level Variance
	High Gray Level Emphasis
	Large Dependence Emphasis
	Large Dependence High Gray Level Emphasis
	Large Dependence Low Gray Level Emphasis
	Low Gray Level Emphasis
	Small Dependence Emphasis
	Small Dependence High Gray Level Emphasis
	Small Dependence Low Gray Level Emphasis
Gray-Level Run Length Matrix (16)	Gray Level Non Uniformity
	Gray Level Non Uniformity Normalized
	Gray Level Variance
	High Gray Level Run Emphasis
	Long Run Emphasis
	Long Run High Gray Level Emphasis
	Long Run Low Gray Level Emphasis
	Low Gray Level Run Emphasis
	Run Entropy
	Run Length Non Uniformity
	Run Length Non Uniformity Normalized
	Run Percentage

	Run Variance
	Short Run Emphasis
	Short Run High Gray Level Emphasis
	Short Run Low Gray Level Emphasis
Gray-Level Size Zone Matrix (16)	Gray Level Non Uniformity
	Gray Level Non Uniformity Normalized
	Gray Level Variance
	High Gray Level Zone Emphasis
	Large Area Emphasis
	Large Area High Gray Level Emphasis
	Large Area Low Gray Level Emphasis
	Low Gray Level Zone Emphasis
	Size Zone Non Uniformity
	Size Zone Non Uniformity Normalized
	Small Area Emphasis
	Small Area High Gray Level Emphasis
	Small Area Low Gray Level Emphasis
	Zone Entropy
	ZonePercentage
	Zone Variance
Neighbourhood Gray-Tone Difference Matrix (5)	Busyness
	Coarseness
	Complexity
	Contrast
	Strength

Supplementary Table S2. Comparison of Model Performances in the Training (a) and Validation (b) Sets, with p-values from the McNemar's Test.

Model	Radiomic	Combined Pre-Treatment	Post – Treatment Clinical	Combined Post-Treatment
Radiomic	1.00	0.25	0.07	0.25
Combined Pre-Treatment		1.00	0.18	1.00
Post – Treatment Clinical			1.00	0.18
Combined Post-Treatment				1.00

a.

Model	Radiomic	Combined Pre-Treatment	Post – Treatment Clinical	Combined Post-Treatment
Radiomic	1.00	0.50	0.25	1.00
Combined Pre-Treatment		1.00	0.50	0.50
Post – Treatment Clinical			1.00	0.25
Combined Post-Treatment				1.00

b.

Supplementary Figures

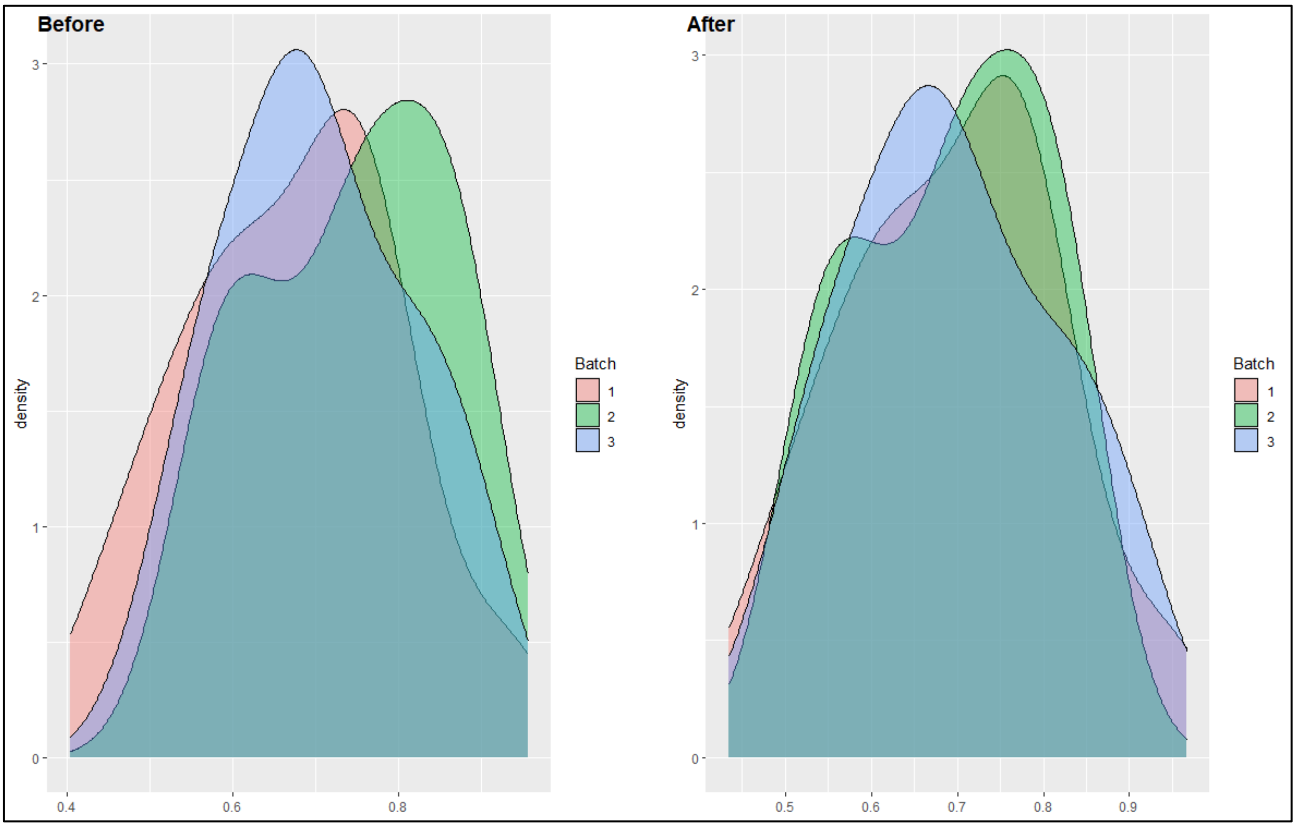

Supplementary Figure S1. Effect of ComBat-based feature harmonization across different scanners and acquisition protocols. The three patient groups included for harmonization are: 1) patients who underwent a contrast-enhanced dynamic sequence at 1.5 T (Batch 1), 2) patients who underwent a contrast-enhanced dynamic sequence at 3 T (Batch 2), and 3) patients who underwent a DCE-MRI at 3 T (Batch 3).

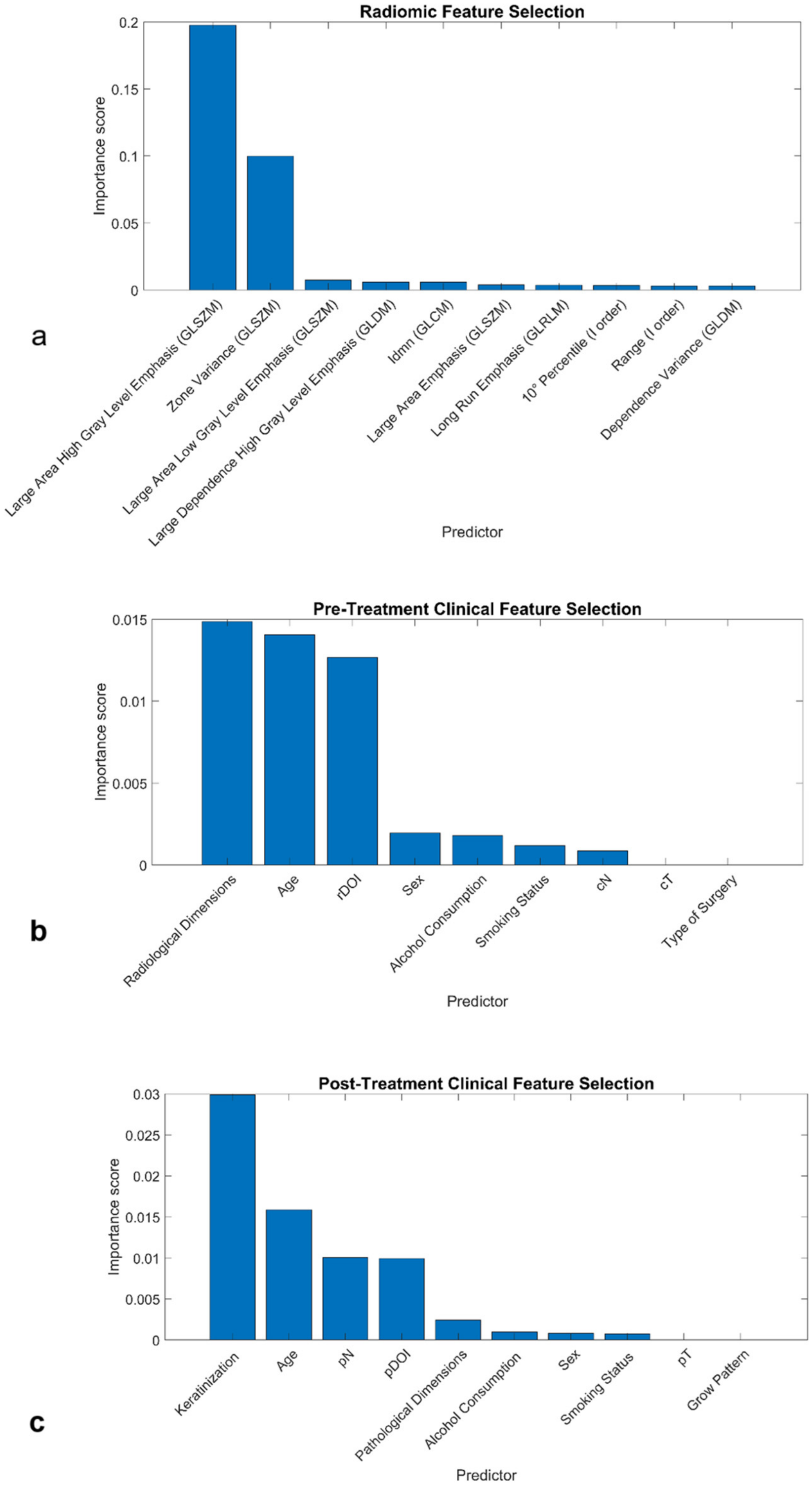

Supplementary Figure S2. Feature Selection using the AdaBoost Algorithm for Radiomic-only Features (a), Pre-Treatment Clinical Features (b), and Post-Treatment Clinical Features (c).

TRAINING SET

True Class \ Predicted Class	No	Loco-regional Recurrence
No	32	11
Loco-regional Recurrence	7	36

VALIDATION SET

True Class \ Predicted Class	No	Loco-regional Recurrence
No	13	5
Loco-regional Recurrence	2	7

a

TRAINING SET

True Class \ Predicted Class	No	Loco-regional Recurrence
No	32	11
Loco-regional Recurrence	9	34

VALIDATION SET

True Class \ Predicted Class	No	Loco-regional Recurrence
No	12	6
Loco-regional Recurrence	2	7

b

TRAINING SET

True Class \ Predicted Class	No	Loco-regional Recurrence
No	34	9
Loco-regional Recurrence	15	28

VALIDATION SET

True Class \ Predicted Class	No	Loco-regional Recurrence
No	12	6
Loco-regional Recurrence	3	6

c

TRAINING SET

True Class \ Predicted Class	No	Loco-regional Recurrence
No	32	11
Loco-regional Recurrence	9	34

VALIDATION SET

True Class \ Predicted Class	No	Loco-regional Recurrence
No	13	5
Loco-regional Recurrence	2	7

d

Supplementary Figure S3. Confusion matrices for the training and validation sets of each proposed model: Radiomic Model (a); Combined Pre-Treatment Model (b), Post-Treatment Clinical Model (c) and Combined Post-Treatment Model (d).
